# Assessing the effects of medium-chain fatty acids and fat sources on PEDV infectivity

**DOI:** 10.1093/tas/txz179

**Published:** 2019-11-29

**Authors:** Roger A Cochrane, Steve S Dritz, Jason C Woodworth, Charles R Stark, Marut Saensukjaroenphon, Jordan T Gebhardt, Jianfa Bai, Richard A Hesse, Elizabeth G Poulsen, Qi Chen, Phillip C Gauger, Rachel J Derscheid, Jianqiang Zhang, Michael D Tokach, Rodger G Main, Cassandra K Jones

**Affiliations:** 1 Department of Animal Sciences and Industry, Kansas State University, Manhattan, KS; 2 Department of Diagnostic Medicine/Pathobiology, College of Veterinary Medicine, Kansas State University, Manhattan, KS; 3 Department of Grain Sciences and Industry, Kansas State University, Manhattan, KS; 4 Veterinary Diagnostic Lab, College of Veterinary Medicine, Kansas State University, Manhattan, KS; 5 Veterinary Diagnostic & Production Animal Medicine, College of Veterinary Medicine, Iowa State University, Ames, IA

**Keywords:** fat source, medium-chain fatty acids, porcine epidemic diarrhea virus, swine

## Abstract

The overall objective of this study was to compare the efficacy of medium-chain fatty acids (MCFA) to other common fat sources to minimize the risk of porcine epidemic diarrhea virus (PEDV) cross-contamination in a pig bioassay. Treatments were feed with mitigants inoculated with PEDV after application and were: 1) positive control with no chemical treatment; 2) 0.325% commercially available formaldehyde-based product; 3) 1% blend of 1:1:1 caproic (C6), caprylic (C8), and capric acids (C10) and applied with an aerosolizing nozzle; 4) treatment 3 applied directly into the mixer without an aerosolizing nozzle; 5) 0.66% caproic acid; 6) 0.66% caprylic acid; 7) 0.66% capric acid; 8) 0.66% lauric acid; 9) 1% blend of 1:1 capric and lauric acids; 10) 0.3% commercially available dry C12 product; 11) 1% canola oil; 12) 1% choice white grease; 13) 2% coconut oil; 14) 1% coconut oil; 15) 2% palm kernel oil; 16) 1% palm kernel oil; 17) 1% soy oil and four analysis days (0, 1, 3, and 7 post inoculation) as well as 1 treatment of PEDV-negative feed without chemical treatment. There was a treatment × day interaction (*P* < 0.002) for detectable PEDV RNA. The magnitude of the increase in Ct value from d 0 to 7 was dependent upon the individual treatments. Feed treated with individual MCFA, 1% MCFA blend, or commercial-based formaldehyde had fewer (*P* < 0.05) detectable viral particles than all other treatments. Commercial-based formaldehyde, 1% MCFA, 0.66% caproic, 0.66% caprylic, and 0.66% capric acids had no evidence of infectivity 10-d old pig bioassay, while there was no evidence the C12 commercial product or longer chain fat sources inhibited PEDV infectivity. Interestingly, pigs given the coconut oil source with the highest composition of caprylic and capric only showed signs of infectivity on the last day of bioassay. These data suggest some MCFA have potential for reducing post feed manufacture PEDV contamination.

## INTRODUCTION

Porcine epidemic diarrhea virus (PEDV) is known to be spread primarily by fecal-oral contamination, but controlled and epidemiological research has shown PEDV can be spread by complete feed and ingredients (Dee et al., 2014; Pasick et al., 2014; Schumacher et al., 2016). Fecal contamination in the feed supply chain may enter with ingredients or from cross-contamination during the manufacturing, transportation, and storage of feed (Cochrane et al., 2016a). Thermal processing helps reduce the risk of this transmission but is a point-in-time mitigant that offers no residual protection from post-processing cross-contamination (Cochrane et al., 2017). Biosecurity can limit this cross-contamination but is challenging to implement across the feed manufacturing and delivery industry (Cochrane et al., 2016a).

Research has demonstrated mitigation additives can reduce the likelihood of viral contamination in a feed matrix. Additionally, mitigation additives can provide residual activity (Cochrane et al., 2016b; Dee et al., 2016; Trudeau et al., 2016). For example, formaldehyde-based products are highly effective against PEDV. However, they are not labeled for its control (Food and Drug Administration, 2017), and can be perceived negatively by consumers (Jones, 2011). An alternative mitigation additive is medium-chain fatty acids (MCFA).

Initial research has demonstrated that a 2 and 1% inclusion of a 1:1:1 ratio of caproic, caprylic, and capric acids is as effective as formaldehyde-based products at reducing the quantity and infectivity of PEDV RNA in a complete swine diet (Cochrane et al., 2015, 2016b). Currently, it is unknown, if the specific ratio of MCFA needs to be utilized to reduce PEDV. Furthermore, it is not known if a specific fatty acid is driving the response to the MCFA blend, or if it is simply an effect of added fat in the diet. The MCFA blend has also been applied using a special aerosolized nozzle to reduce droplet size which is a similar process used for the application of commercial formaldehyde-based products (Cochrane et al., 2015, 2016b). This would require special equipment to be installed into feed mills thus making it more difficult to utilize MCFA in complete swine feed. It is unknown if direct addition to the mixer would be sufficient for MCFA application making the use of MCFA a more realistic approach.

Fat sources used in the swine industry also contain MCFA and medium-chain triglycerides (MCT). Common fats used in the United States swine industry include choice white grease and soy oil which contain low levels of MCT and MCFA. However, other countries utilize fat sources such as coconut oil and palm kernel oil which contain higher levels of MCT and MCFA. However, it is not known how effective the products would be as a PEDV reduction strategy because of the MCFA being contained within the MCT molecules. These fat sources would be significantly lower cost than the MCFA sources and are widely available. Therefore, the overall objective of this study was to compare the efficacy of MCFA to other common fat sources to minimize the risk of PEDV cross-contamination in a pig bioassay.

## MATERIALS AND METHODS

The Iowa State University Institutional Animal Care and Use Committee approved the pig bioassay protocol. In order to evaluate the use of chemical treatments and fat sources on PEDV survival, a corn and soybean meal-based swine diet was manufactured at the Kansas State University O.H. Kruse Feed Technology Innovation Center, Manhattan (Table 1).

**Table 1.** Diet composition

Item	Negative control
Ingredient, %	
Corn	79.30
Soybean meal, 46.5% CP	15.70
Choice white grease	1.00
Monocalcium phosphate	1.40
Limestone, ground	1.15
Salt	0.50
L-Threonine	0.03
Trace mineral premix^*a*^	0.15
Sow add pack^*b*^	0.50
Vitamin premix^*c*^	0.25
Phytase^*d*^	0.02
Total	100.00
Formulated analysis, %	
Dry matter	91.4
Crude protein	17.1
Crude fiber	3.7
Ether extract	3.5
Ca	0.78
P	0.52

^*a*^Each kilogram contains 26.4 g Mn, 110 g Fe, 110 g Zn, 11g Cu, 198 mg I, and 198 mg Se.

^*b*^Each kilogram contains 220,000 mg choline, 88 mg biotin, 660 mg folic acid, 1,980 mg pyridoxine.

^*c*^Each kilogram contains 4,400,000 IU vitamin A, 660,000 IU vitamin D_3_, 17,600 IU vitamin E, 1,760 mg menadione, 3,300 mg riboflavin, 11,000 mg pantothenic acid, 19,800 mg niacin, 15.4 mg vitamin B_12_.

^*d*^High Phos 2700 GT, DSM Nutritional Products, Parsippany, NJ.

### Treatment Application

The diet was either left untreated (control) or was mixed with different mitigants prior to inoculation with PEDV to test residual mitigation capability. The 18 treatments were 1) positive control without mitigation treatment but inoculated with PEDV; 2) 0.325% commercial-based formaldehyde product (Kemin Industries, Des Moines, IA; Sal CURB); 3) 1% blend of 1:1:1 caproic, caprylic, and capric acids and applied with an aerosolizing nozzle (MCFA aerosolized); 4) treatment 3 applied directly into the mixer without an aerosolizing nozzle (MCFA non-aerosolized); 5) 0.66% caproic acid; 6) 0.66% caprylic acid; 7) 0.66% capric acid; 8) 0.66% lauric acid; 9) 1% blend of 1:1 capric and lauric acids; 10) 0.3% commercially available dry C12 product (FRA C12, Framelco, Raamsdonksveer, Netherlands); 11) 1% canola oil; 12) 1% choice white grease; 13) 2% coconut oil; 14) 1% coconut oil; 15) 2% palm kernel oil; 16) 1% palm kernel oil;17) 1% soy oil and 1 treatment of PEDV-negative feed without mitigant treatment. Treatments 5–8 were included at rates derived from the original 2% inclusion of the 1:1:1 ratio of caproic, caprylic, and capric acids utilized from Cochrane et al. (2015). Thus a 1:1:1 ratio of three products included to 2% would be equal to 0.66% of each product. Choice white grease, soy oil, canola oil, palm kernel oil, and coconut oil were analyzed for their fatty acid profiles at the Agricultural Experiment Station Chemical Laboratories, University of Missouri-Columbia, College of Agriculture, Food and Natural Resources using AOAC method 996.06 (1997).

All treatments were added on a weight to weight basis and mixed using laboratory-scale paddle mixers (Cabela’s Inc., Sidney, NE) that had been validated for mixing efficiency. Mixers were sanitized between treatments. The commercially based formaldehyde and MCFA aerosolized treatment were mixed using an air atomizing nozzle to reduce the droplet size of the liquid treatments. The remaining treatments were added directly to the mixer. Each dietary treatment was made in triplicate batches in a clean mixer. After each treatment was mixed, 22.5 g of feed was collected from eight different locations within the mixer to create a composite subsample and then added to 1 of 12–250 mL high-density polyethylene, square, wide-mouth bottles (Thermo Fisher Scientific, Waltham, MA) per treatment (3 replications per treatment × 4 analysis days = 12 bottles per treatment for inoculation).

### Inoculation

The U.S. PEDV prototype strain cell culture isolate USA/IN/2013/19338, passage 8 (PEDV19338) was isolated, propagated, and titrated in Vero cells (ATCC CCL-81) as described by Chen et al. (2014). The stock virus titer contained 4.5 × 10^6^ TCID_50_/mL, and was diluted to 10^5^ TCID_50_/mL using cell culture media to create the viral inoculum. In all bottles except the negative controls, 2.5 mL of inoculum was added to the 22.5 g of each feed treatment. Each bottle was thoroughly shaken to ensure equal dispersion of the virus, resulting in each inoculated bottle containing feed with a PEDV concentration of 10^4^ TCID_50_/g.

### RT-qPCR Analysis

Samples were either aliquoted for viral RNA expression of PEDV via qRT-PCR immediately (d 0) or stored at room temperature for analysis on d 1, 3, and 7 post inoculation. For analysis, 100 mL of chilled PBS was added to each bottle, which were then shaken and chilled overnight at 4°C. Three samples of supernatant were then collected, including two PCR samples and a bioassay sample, and stored at −80°C until the end of the experiment. Samples were analyzed by a duplex real-time RT-PCR (RT-qPCR) targeting the Spike gene of PEDV according to Huss et al. (2017). Results are reported in cycle threshold (Ct). A higher Ct value means less genetic material was present.

### Bioassay Analysis

A total of 15 treatments were selected for the bioassay and were the d 0 post-inoculation: 1) negative control with no PEDV and no mitigant treatment; or 2) positive control with PEDV and no chemical treatment; or from d 1 post-inoculation: 3) positive control with PEDV and no chemical treatment; 4) 0.325% commercial-based formaldehyde; 5) 1% MCFA non-aerosolized; 6) 0.66% caproic acid; 7) 0.66% caprylic acid; 8) 0.66% capric acid; 9) 0.66% lauric acid; 10) 0.3% FRA C12; 11) 1% canola oil; 12) 1% choice white grease; 13) 1% coconut oil; 14) 1% palm kernel oil; 15) 1% soy oil.

The bioassay procedure was carried out using the same procedures and the same pig source used in previously reported studies (Schumacher et al., 2016, 2018; Gebhardt et al., 2018b). In brief, a total of 45 crossbred, 10 d-old pigs of mixed sex were sourced from a single commercial, crossbred farrow-to-wean herd with no prior exposure to PEDV. The Iowa State University Veterinary Diagnostic Laboratory confirmed all pigs negative for PEDV, porcine delta coronavirus, and transmissible gastroenteritis virus by fecal swab. Blood serum analysis further confirmed no prior PEDV exposure through analysis of PEDV antibodies by an indirect fluorescent antibody assay. Pigs were allowed 2 d of adjustment prior to beginning the bioassay. A total of 45 pigs were individually housed, and 1 pig challenged via oral gavage for each of the 3 replicate batches of feed. Oral gavage methods have been previously reported by Thomas et al. (2015). Rectal swabs were collected on d-2, 0, 2, 4, 6, and 7 post inoculation (dpi) from all pigs and tested for PEDV RNA by qRT-PCR. Following humane euthanasia at 7 dpi, small intestine, cecum, and colon samples were collected at necropsy along with an aliquot of cecal contents as described by Schumacher et al. (2018). A negative bioassay was determined if all rectal swabs and cecum contents had non-detectable levels of PEDV. If any samples had detectable RNA, the result was considered a positive bioassay.

### Statistical Analysis

Data of the main effects of treatment, day, and the interaction were analyzed as a completely randomized design using PROC GLIMMIX in SAS v9.4 (SAS Institute, Inc., Cary, NC). Results were considered significant if *P* ≤ 0.05 and marginally significant if 0.05 < *P* ≤ 0.10. The PEDV negative control with no PEDV and no mitigation treatment was not included in the statistical analysis as the samples were only analyzed on d 0 to show that no PEDV RNA was detected in the complete feed.

## RESULTS

### Fatty Acid Analysis

Fatty acid profiles for choice white grease, soy oil, canola oil, palm kernel oil, and coconut oil are displayed in Table 2. Coconut oil and palm kernel oil provided the greatest concentration of MCFA.

**Table 2.** Fatty acid profile for each fat source^*a*^

Item	Soy oil	Coconut oil	Canola oil	Choice white grease	Palm kernel oil
Caproic C6:0	0.0	0.3	<0.1	<0.1	0.1
Caprylic C8:0	0.0	5.1	0.0	<0.1	2.5
Capric C10:0	0.0	5.2	<0.1	0.1	2.9
Lauric C12:0	0.0	46.8	<0.1	0.1	45.8
Myristic (14:0)	0.1	19.5	0.1	1.4	16.6
Myristoleic (9c-14:1)	0.0	0.0	0.0	<0.1	0.0
C15:0	<0.1	<0.1	<0.1	0.1	<0.1
Palmitic (16:0)	10.8	10.2	3.9	22.4	9.2
Palmitoleic (9c-16:1)	0.1	<0.1	0.2	2.5	<0.1
Margaric (17:0)	0.1	<0.1	0.1	0.4	<0.1
10c-17:1	0.0	0.0	0.0	0.0	0.0
Stearic (18:0)	3.9	3.3	1.7	9.7	2.4
Elaidic (9t-18:1)	0.0	<0.1	<0.1	0.0	<0.1
Oleic (9c-18:1)	20.2	7.3	59.1	40.9	17.2
Vaccenic (11c-18:1)	1.5	0.2	3.0	2.8	0.0
Linoleic (18:2n6)	53.6	1.8	19.0	14.2	2.8
Linolenic (18:3n3)	7.9	<0.1	8.8	0.5	<0.1
Stearidonic (18:4n3)	0.0	0.0	0.0	0.0	0.0
Arachidic (20:0)	0.3	0.1	0.6	0.2	0.1
Gonodic (20:1n9)	0.2	0.1	1.6	0.9	0.11
C20:2	0.1	0.0	0.1	0.7	<0.1
Homo-a-linolenic(20:3n3)	0.0	0.0	0.0	0.0	<0.1
Arachidonic [20:4n6]	0.0	0.0	0.0	0.3	<0.1
3n-Arachidonic (20:4n3)	0.0	0.0	0.0	0.0	<0.1
EPA (20:5n3)	0.0	0.0	0.0	<0.1	<0.1
Behenoic (22:0)	0.4	<0.1	0.3	<0.1	<0.1
Erucic [22:1n9]	<0.1	0.0	<0.1	<0.1	<0.1
Clupanodonic (22:5n3)	0.0	0.0	0.0	<0.1	<0.1
DHA (22:6n3)	0.0	0.0	0.0	<0.1	<0.1
Lignoceric (24:0)	0.1	<0.1	0.2	<0.1	0.1
Nervonic (24:1n9)	0.0	0.0	0.2	<0.1	0.0

^*a*^Expressed as percent of total fat. W/W% = grams per 100 g of sample. Results are expressed on an “as is” basis unless otherwise indicated.

### qRT-PCR Results

No PEDV RNA was detected in the feed sample without PEDV or mitigation treatment. There was a treatment × day interaction (*P* = 0.0002) for detectable PEDV RNA (Table 3). The MCFA treatments of 1% MCFA (aerosolized and not aerosolized), 0.66% caproic, and 0.66% caprylic each differed (*P* < 0.05) from the commercial formaldehyde treatment on d 0 showing a greater magnitude of initial reduction of detectable PEDV RNA. However, by d 7, 0.66% caproic, and 0.66% caprylic were similar (*P* > 0.05) to the commercial formaldehyde demonstrating that after d 0, the magnitude of decrease of the detectable PEDV RNA was greater in the commercial formaldehyde product. This goes to show that the magnitude of the increase in Ct value on the initial analysis day and from d 0 to 7 was dependent upon the individual treatments. For example, an 8.7 increase in Ct was noted in the commercial-based formaldehyde product compared with a 3.7 Ct increase in choice white grease by d 7.

**Table 3.** Effect of treatment × day post inoculation on PEDV detection using RT-PCR cycle threshold^*a,b*^

	Day					Treatment × day
Item	0	1	3	7	SEM	*P*
PEDV positive	28.3^wvxyz^	29.7^rstuv^	31.3^nopq^	32.7^klmn^	0.5239	0.0002
0.325% Commercial formaldehyde^*c*^	28.7^uvwxy^	33.0^jklm^	35.0^fgh^	37.3^cd^		
1% MCFA (aerosolized)^*d*^	33.3^ijkl^	36.3^def^	38.3^bc^	39.0^ab^		
1% MCFA (non-aerosolized)^*e*^	34.3^ghij^	38.3^bc^	37.0^cde^	40.0^a^		
0.66 % Caproic acid	33.7^hijk^	35.0^fgh^	36.3^def^	37.0^cde^		
0.66% Caprylic acid	34.3^ghij^	35.7^efg^	38.0^bc^	37.3^cd^		
0.66% Capric acid	29.3^stuvw^	30.7^opqrs^	34.0^ghij^	35.3^fg^		
0.66 % Lauric acid	28.3^vwxyz^	30.7^opqrs^	32.7^klmn^	34.7^ghi^		
1% Capric:Lauric acids^*f*^	29.0^tuvwx^	31.7^mnop^	34.3^ghij^	34.3^ghij^		
0.3% FRA C12^*g*^	28.0^wxyz^	30.7^opqrs^	31.7^mnop^	33.7^hijk^		
1% Canola oil	27.0^z^	30.7^opqrs^	31.0^opqr^	31.7^mnop^		
1% Choice white grease	28.3^vwxyz^	30.0^qrstu^	30.7^opqrs^	32.0^lmno^		
1% Coconut oil	28.0^wxyz^	30.3^pqrst^	31.3^nopq^	32.7^klmn^		
2% Coconut oil	27.3^yz^	29.3^stuvw^	29.7^rstuv^	32.7^klmn^		
1% Palm kernel oil	27.7^xyz^	30.0^qrstu^	31.0^opqr^	33.0^klmn^		
2% Palm kernel oil	27.3^yz^	29.7^rstuv^	30.3^pqrst^	33.0^jklm^		
1% Soy oil	27.7^xyz^	30.0^qrstu^	30.3^pqrst^	32.0^lmno^		

^*a*^A complete swine diet was first treated with 18 treatments and then inoculated with PEDV to mimic post-processing contamination. Once the inoculation was complete, the samples were analyzed on d 0, 1, 3, and 7 post inoculation for detectable PEDV RNA. Means presented are the interactive means of each treatment by analysis day and represented by *n* of 3. The PEDV negative treatment was analyzed on d 0 to verify that no PEDV was present in the feed. However, after this determination, it was not included in the statistical analysis as it was only analyzed on d 0.

^*b*^Cycle threshold required to detect the genetic material. A higher Ct value means less genetic material present. Means within a column and row lacking a common superscript differ (*P* < 0.05).

^*c*^Sal Curb, Kemin Industries, Des Moines, IA.

^*d*^MCFA blend of 1:1:1 ratio of caproic, caprylic, and capric acids aerosolized into the mixer via an air atomizing nozzle.

^*e*^MCFA blend of 1:1:1 ratio of caproic, caprylic, and capric acids added directly into the mixer with no atomizing nozzle.

^*f*^1:1 ratio of capric and lauric acids.

^*g*^Framelco, Raamsdonksveer, Netherlands.

As time increased, each analysis day had less (*P* < 0.0001) detectable PEDV RNA compared with each previous analysis day (Table 4). Mitigation treatment also impacted (*P* < 0.0001) the quantity of detectable PEDV RNA. The MCFA blends (1% MCFA and 1% capric:lauric), caproic acid, caprylic acid, capric acid, lauric acid, and commercial-based formaldehyde reduced (*P* < 0.05) the quantity of detectable PEDV RNA compared with the positive control. There was no evidence delivery method impacted (*P* > 0.05) Ct value of the 1% MCFA blend. Also, there was no evidence the feed with FRA C12, choice white grease, soy oil, canola oil, palm kernel oil, and coconut oil, regardless of inclusion level had a different (*P* > 0.05) Ct value compared with the PEDV positive control feed.

**Table 4.** Main effects of day and treatment on PEDV detection using qRT-PCR^*a*^

Item	Ct^*b*^	SEM	*P*
Analysis day^*c*^		0.130	<0.0001
d 0	29.5^a^		
d 1	31.9^b^		
d 3	33.1^c^		
d 7	34.6^d^		
Mitigation treatment^*d*^		0.262	<0.0001
PEDV negative	> 40.0		
PEDV positive	30.5^gh^		
0.325% Commercial formaldehyde^*e*^	33.5^d^		
1% MCFA (aerosolized)^*f*^	36.8^ab^		
1% MCFA (non-aerosolized)^*g*^	37.4^a^		
0.66 % Caproic acid	35.5^b^		
0.66% Caprylic acid	36.3^c^		
0.66% Capric acid	32.4^e^		
0.66 % Lauric acid	31.6^f^		
1% Capric:lauric acids^*h*^	32.3^e^		
0.3% FRA C12^*i*^	31.0^fg^		
1% Choice white grease	30.3^hi^		
1% Canola oil	30.1^hi^		
1% Coconut oil	30.6^gh^		
2% Coconut oil	29.8^i^		
1% Palm kernel oil	30.3^ghi^		
2% Palm kernel oil	30.1^hi^		
1% Soy oil	30.0^hi^		

^*a*^A complete swine diet was first treated with 18 treatments and then inoculated with PEDV to mimic post-processing contamination. Once the inoculation was complete, the samples were analyzed on d 0, 1, 3, and 7 post inoculation for detectable PEDV RNA. Means presented in the table are for the main effect of day and treatments.

^*b*^Cycle threshold required to detect the genetic material. A higher Ct value means less genetic material present. The cycle threshold of ≥ 40 was considered negative for the presence of PEDV RNA Means within analysis day and mitigation treatment lacking a common superscript differ (*P* < 0.05).

^*c*^Main effect of analysis day on PEDV RNA detectability. Each analysis d is represented by an *N* of 51.

^*d*^Main effect of mitigation treatment on PEDV RNA detectability. Each treatment besides the PEDV negative treatment is represented by an *N* of 12. The PEDV negative treatment was analyzed on d 0 to verify that no PEDV was present in the feed. However, after this determination, it was not included in the statistical analysis as it was only analyzed on d 0. The PEDV negative mean is represented by and *N* of 3.

^*e*^Sal Curb, Kemin Industries, Des Moines, IA.

^*f*^MCFA blend of 1:1:1 ratio of caproic, caprylic, and capric acids aerosolized into the mixer via an air atomizing nozzle.

^*g*^MCFA blend of 1:1:1 ratio of caproic, caprylic, and capric acids added directly into the mixer with no atomizing nozzle.

^*h*^1:1 ratio of capric and lauric acids

^*i*^Framelco, Raamsdonksveer, Netherlands.

### Bioassay Results

There was no evidence of infection in pigs challenged with the PEDV-negative feed. However, pigs receiving the PEDV-infected feed without chemical mitigation had evidence of PEDV infectivity. Treatments without detectable evidence of PEDV infection were fed treated with commercial-based formaldehyde, MCFA blend, caproic acid, caprylic acid, and capric acid (Table 5). Pigs challenged with feed containing lauric acid, FRA C12, choice white grease, or any of the vegetable oil sources had evidence of PEDV infectivity. Notably, pigs receiving the coconut oil treatment had no detectable PEDV RNA until d 7 after challenge.

**Table 5.** Effects of treatment on PEDV infectivity measured by pig fecal swabs and cecum content by qRT-PCR analysis^*a*^

	PEDV N-gene Real Time-PCR, cycle threshold (Ct)						
		Fecal swabs						Cecum contents^*d*^
Item	Feed Ct^*b*^	0 dpi^*c*^	2 dpi	4 dpi	6 dpi	7 dpi	7 dpi
d 0^*e*^							
PEDV negative	> 40.0	*---* ^*f*^	---	---	---	---	> 45.0
PEDV positive	28.3	---	-- +	+++	+++	+++	22.2
d 1^*g*^							
PEDV positive	29.7	---	- ++	+++	+++	+++	20.9
0.325% Commercial formaldehyde^*h*^	33.0	---	---	---	---	---	> 45.0
1% MCFA (non-aerosolized)^*i*^	38.3	---	---	---	---	---	> 45.0
0.66 % Caproic acid	35.0	---	---	---	---	---	> 45.0
0.66% Caprylic acid	35.7	---	---	---	---	---	> 45.0
0.66% Capric acid	30.7	---	---	---	---	---	> 45.0
0.66 % Lauric acid	30.7	---	---	+++	+++	+-+	28.4
0.3% FRA C12^*j*^	30.7	---	---	+++	+++	+++	30.2
1% Canola oil	30.7	---	---	+++	+++	+++	20.3
1% Choice white grease	30.0	---	---	+++	+++	+++	15.3
1% Coconut oil	30.3	---	---	---	---	+-+	42.1
1% Palm kernel oil	30.0	---	---	+++	+++	+++	22.1
1% Soy oil	30.0	---	---	+++	+++	+++	24.0

^*a*^An initial tissue culture containing 10^6^ TCID_50_/mL PEDV was diluted to 10^5^ TCID_50_/mL PEDV. Each treatment was inoculated with the 10^5^ TCID_50_/mL PEDV resulting in 10^4^ TCID_50_/g PEDV inoculated feed matrix. Three feed samples per day and treatment were collected and diluted in PBS. The supernatant from each sample was then collected for pig bioassay. The supernatant was administered one time via oral gavage on d 0 to each of three pigs per treatment (10 mL per pig). Thus, the cecum contents are represented by a mean of 3 pigs per treatment. Pigs were inoculated at d 12 age.

^*b*^A cycle threshold (Ct of > 40) was considered negative for the presence of PEDV RNA. Feed Ct analysis via qRT-PCR was carried out at Kansas State University. Values are from each analysis day by treatment interaction.

^*c*^Day post inoculation.

^*d*^A cycle threshold (Ct of > 45) was considered negative for the presence of PEDV RNA. Cecum content analysis via qRT-PCR was carried out at Iowa State University Veterinary Diagnostic Laboratory at necropsy of the bioassay. Each value is represented by an *n* of 3 pigs.

^*e*^D 0 samples are represented from the d 0 analysis day and collected during the qRT-PCR analysis. The samples were collected and kept at −80°C until given to a 12 d old pig via oral gavage.

^*f*^ In each instance a (–) signals a negative pig in the bioassay and a (+) represents a positive fecal swab in the bioassay. Each day post inoculation within each treatment has three symbols within each row and column which represents one of the three pigs in each treatment.

^*g*^D 1 samples are represented from the d 1 analysis day and collected during the qRT-PCR analysis. The samples were collected and kept at −80°C until given to a 12 d old pig via oral gavage.

^*h*^Kemin Industries, Des Moines, IA.

^*i*^MCFA blend of 1:1:1 ratio of caproic, caprylic, and capric acids added directly into the mixer with no atomizing nozzle.

^*j*^Framelco, Raamsdonksveer, Netherlands.

## DISCUSSION

Previous research demonstrating the efficacy of MCFA as a PEDV mitigant had applied commercial-based formaldehyde products and MCFA using an aerosolizing nozzle (Cochrane et al., 2015, 2016b). This system reduces droplet size to increase the surface area of the applied liquids. These systems are expensive to install and require greater maintenance than typical fat application systems. This research demonstrates that an aerosolizing liquid application system is not necessary for MCFA to have maximum efficacy for PEDV mitigation, which is an important consideration for feed mills.

In agreement with previous research, MCFA and formaldehyde-based products reduced the detectable PEDV RNA by qRT-PCR in swine feed (Cochrane et al., 2016b; Dee et al., 2016; Gebhardt et al. 2018a). In general, the 1% MCFA blend performed similar to previous research by Cochrane et al. (2016b). However, this experiment goes further than previous research to identify the most effective components of this MCFA blend. The mitigation success of the 1% MCFA blend is driven by caprylic acid and caproic acid, as they provided the greatest reduction in detectable PEDV (35.5 and 36.3 Ct, respectively) compared with capric acid (32.4 Ct). While, the 0.66% inclusion of any of the three led to a negative bioassay, none had a similar reduction in detectable PEDV of the 1% MCFA blend (37.4 Ct). This suggests there is a possible synergistic effect of the MCFA when in combination with one another, but additional research is necessary to identify the minimum inhibitory concentration of MCFA alone or in combination with various feed matrices.

MCFA have been shown to destabilize the cellular membrane bi-layer of bacteria by incorporating themselves into the lipid bi-layer because of the similar hydrophilic/lipophilic balance (Desbois and Smith, 2010; Kim and Rhee, 2013). This in turn causes pores to be created altering the cellular membrane and leading to cell death (Desbois and Smith, 2010; Kim and Rhee, 2013). Because of this mode of action with bacteria, we hypothesize that the greater efficacy of the blend and shorter MCFA may be due to how these specific fatty acids interact with the lipid and protein components of the envelope of the virus. Specifically, we believe that the relatively neutral pH of MCFA allows these fatty acids to interact with the lipids within the viral envelope. This would then cause pores to be created and lead to destabilization of the viral envelope with an effect similar to the bacterial mode of action. If this mode of action is true, then the viral envelope would not be able to attach to the host and lead to no replication.

Research in other enveloped viruses has demonstrated the success of MCFA as a mitigant (Thormar et al., 1987; Hilmarsson et al., 2006), but this mode of action needs to be confirmed in PEDV. Thormar et al. (1987) suggested that the MCFA are disrupting, and depending upon the MCFA and concentration, disintegrating the viral envelopes. Our hypothesis would then agree with this statement, and potentially describe why the level of detectable genetic material decreases over time at a faster rate when MCFA is included in the diet.

As our working hypothesis is that the MCFA can approach the PEDV envelope and cause destabilization, it was necessary to confirm that this effect is unique to MCFA, and not to other lipids. The natural triglycerides (choice white grease coconut oil, corn oil, palm kernel oil, and soy oil) provided no benefit to reducing viral RNA, and feeds treated with all resulted in infectivity. However, pigs receiving the coconut oil treatment had delayed clinical signs of PEDV. Based on its fatty acid analysis, coconut oil had the greatest MCFA concentrations of the natural fat sources. The total quantity of caproic, caprylic, and capric acids in the final diet were 0.11% or 0.21 in the 1 or 2% coconut oil treatments, respectively. While this is lower than 0.66% of each individual fatty acid or the 1% MCFA blend that demonstrated no infectivity, the small quantity of MCFA in the coconut oil may have led to a delay in PEDV infectivity. Another natural fat source with known levels of MCFA is palm kernel oil, but the concentration of caproic, caprylic, and capric acids was lower than in coconut oil. Treatments with palm kernel oil had no protection from PEDV. Furthermore, the MCFA in either the coconut or palm kernel oil treatments were presumably MCT. It is thought that longer MCFA, such as lauric and myristic acids, are too lipophilic to approach the PEDV cellular membrane, while those bound in a triglyceride need hydrolysis from lipase to carry on its mode of action.

In summary, this research suggests a specialized aerosolized nozzle is not necessary to apply MCFA for PEDV mitigation. It was also demonstrated that 0.66% caproic, 0.66% caprylic, and 0.66% capric acids have similar efficacy as commercial grade formaldehyde or the 1% MCFA blend when placed into a 10 d old swine bioassay. Interestingly, pigs given the coconut oil source with the highest composition of caprylic and capric only showed signs of infectivity on the last day of bioassay. Unfortunately, it was also shown that these MCFA are more effective at mitigating PEDV than longer chain fatty acids or triglycerides. However, further research needs to be carried out to evaluate alternative MCFA combinations, and MCFA concertation levels against PEDV.
